# Emerging roles of Myc in stem cell biology and novel tumor therapies

**DOI:** 10.1186/s13046-018-0835-y

**Published:** 2018-07-27

**Authors:** Go J. Yoshida

**Affiliations:** Department of Pathological Cell Biology, Medical Research Institute, Tokyo Medical and Dental University, 1-5-45 Yushima, Bunkyo-ku, Tokyo, 113-8510 Japan

**Keywords:** Cancer stem cells, CD44 variant, Drug-repositioning, Epigenetics, FBW7, Induced pluripotent stem cells, Metabolic reprogramming, Myc family, Neuroendocrine tumors, Ubiquitination

## Abstract

The pathophysiological roles and the therapeutic potentials of Myc family are reviewed in this article. The physiological functions and molecular machineries in stem cells, including embryonic stem (ES) cells and induced pluripotent stem (iPS) cells, are clearly described. The c-Myc/Max complex inhibits the ectopic differentiation of both types of artificial stem cells. Whereas c-Myc plays a fundamental role as a “double-edged sword” promoting both iPS cells generation and malignant transformation, L-Myc contributes to the nuclear reprogramming with the significant down-regulation of differentiation-associated genetic expression. Furthermore, given the therapeutic resistance of neuroendocrine tumors such as small-cell lung cancer and neuroblastoma, the roles of N-Myc in difficult-to-treat tumors are discussed. N-Myc-driven neuroendocrine tumors tend to highly express NEUROD1, thereby leading to the enhanced metastatic potential. Importantly enough, accumulating evidence strongly suggests that c-Myc can be a promising therapeutic target molecule among Myc family in terms of the biological characteristics of cancer stem-like cells (CSCs). The presence of CSCs leads to the intra-tumoral heterogeneity, which is mainly responsible for the therapeutic resistance. Mechanistically, it has been shown that Myc-induced epigenetic reprogramming enhances the CSC phenotypes. In this review article, the author describes two major therapeutic strategies of CSCs by targeting c-Myc; Firstly, Myc-dependent metabolic reprogramming is closely related to CD44 variant-dependent redox stress regulation in CSCs. It has been shown that c-Myc increases NADPH production via enhanced glutaminolysis with a finely-regulated mechanism. Secondly, the dormancy of CSCs due to FBW7-depedent c-Myc degradation pathway is also responsible for the therapeutic resistance to the conventional anti-tumor agents, the action points of which are largely dependent on the operation of the cell cycle. That is why the loss-of-functional mutations of *FBW7* gene are expected to trigger “awakening” of dormant CSCs in the niche with c-Myc up-regulation. Collectively, although the further research is warranted to develop the effective anti-tumor therapeutic strategy targeting Myc family, we cancer researchers should always catch up with the current advances in the complex functions of Myc family in highly-malignant and heterogeneous tumor cells to realize the precision medicine.

## Background

c-Myc, N-Myc, and L-Myc encoded by the proto-oncogene family are essential transcription factors which belong to the superfamily of basic helix-loop-helix (bHLH) DNA-binding proteins. These three major members of Myc family are involved in the fundamental normal cellular phenomena including metabolism, cellular division, differentiation, and cell death [1–4]. It is widely accepted that, after the formation of the heterodimer with Myc-associated protein X (Max), Myc activates the transcription by binding to the DNA recognition sequences in the target gene promoters also referred to as E-box region [5, 6]. Since the identification of c-Myc in Burkitt lymphoma approximately 40 years ago [7, 8], the innumerable number of research articles have been published on the pathophysiological contributions of Myc family to maintain the malignant potential [9–12]. The frequency of *c-Myc* mutations was 33.3% at the DNA level (mutations in either the coding sequence or the untranslated regions), and 16.1% at the protein level (nonsynonymous mutations) in diffuse large B-cell lymphoma (DLBCL) [13, 14]. With regard to breast cancer, *c-Myc* amplification is recognized in about one-half of *BRCA1*-mutated tumors, as compared with approximately 20% in sporadic tumors [15]. Notably, genomic and proteomic features associated with Myc and the proximal Myc network (PMN) across more than 30 kinds of cancers in The Cancer Genome Atlas (TCGA) database has recently identified that 28% of all tumor samples have the genetic abnormalities in at least one of the Myc family composed of c-Myc, N-Myc, and L-Myc [16]. Importantly, Max also forms homodimers or heterodimers with its alternative partners, Mad and Mxi-1. These complexes behave as antagonists of Myc/Max through competition for common DNA targets. While Myc/Max heterodimers stimulate transcription, Mad/Max heterodimers repress transcription, in part by recruiting a complex of co-repressors including Sin3 and histone deacetylases (HDAC) [17, 18]. Still, there remain several points to be poorly understood about the complex functions of Myc family in tumor cells. Thus, the pathophysiological roles and the therapeutic potentials of Myc family are reviewed in this article.

## Novel functions of Myc in physiological and artificially-induced stem cells

Both the expression and function of c-Myc are tightly regulated by developmental or mitogenic signals in normal (non-transformed) cells. To better understand the physiological functions of Myc family, many researchers have focused on Myc family expressed in embryonic stem (ES) cell and induced pluripotent stem (iPS) cells. The analysis of embryos derived from the homozygous *c-Myc* mutant ES cell lines reveals the embryonic lethality between 9.5 and 10.5 days of gestation with the significant defects in the hematopoietic and vascular networks [19]. In contrast, the analysis of embryos derived from the homozygous *N-Myc* mutant ES cell lines reveals the embryonic lethality prenatally at approximately 11.5 days of gestation with the disrupted neuroectodermal, heart, and lung development [20–22]. Notably, N-Myc expression analysis of the homozygous *N-Myc* mutant embryonic lung tissues has uncovered that normal level of N-Myc expression is essential for the proliferation of the pulmonary epithelial cells in response to the paracrine signals emanating from the lung mesenchyme [21]. In addition, the conditional knockout of *N-Myc* gene in neural stem cells (NSCs) results in the profound disruption of the normal brain development partially due to the disrupted cellular division of NSCs [23]. Despite of the widely-expressed pattern in the murine embryonic tissues, L-Myc seems to be relatively dispensable for the normal embryonic development compared with c-Myc and N-Myc [24, 25]. Thus, both c-Myc and N-Myc are crucial regulators during the process of normal embryogenesis in that Myc family are essential for the acquisition and maintenance of stem cell properties (also referred to as “stemness”) characterized by self-renewal potential and multi-lineage differentiation ability. However, the potential endogenous functions of Myc family in the regulation of the abilities of self-renewal and pluripotency have not yet been completely clarified.

*Myc*-deficient murine ES cells do not necessarily undergo the terminal stages of differentiation which bring about the fully differentiated progeny, but rather differentiate largely into early progenitor-like cells [26]. The main pluripotency markers such as Oct4 and Nanog are gradually down-regulated upon differentiation, and it is likely that these pluripotency factors are co-expressed with differentiation markers in the initial steps of differentiation. Indeed, the single-cell transcript analysis of human ES cells has revealed the persistence of pluripotency transcriptional products in the differentiated cells, in which various markers of differentiation and pluripotency are co-expressed [27]. Accumulating evidence strongly suggests that the predominant function of Myc family to maintain the pluripotency in mouse ES cells is the potent suppression in the early stage of differentiation. This finding is consistent with the fact that c-Myc does not greatly contribute to the activation of the pluripotency regulators in reprogrammed cells [28]. Of note, the critical role of c-Myc in the regulation of leukemia inhibitory factor (LIF)/signal transduction and activator of transcription 3 (STAT3) signal pathway has been demonstrated in murine ES cells because of LIF-independence due to the constitutively active c-Myc overexpression [29].

**Fig. 1:**
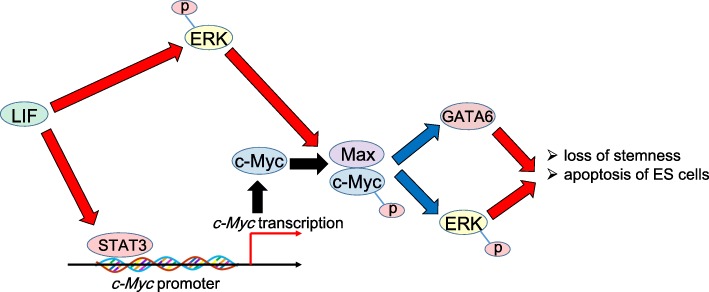
The molecular machinery underlying the maintenance of stemness in ES cells mainly regulated by c-Myc/Max complex. The transcriptional level of proto-oncogene *c-Myc* is promoted by leukemia inhibitory factor (LIF) and the transcriptional factor c-Myc forms a heterodimer with Max after phosphorylated by activated extracellular signal-regulated kinase (ERK). The c-Myc/Max complex suppresses GATA6 expression, and more importantly, forms the negative-feedback mechanism with the inhibition of phosphorylated ERK (p-ERK). Given that GATA6 and p-ERK induce apoptotic cell death of ES cells with caspase activation and reduce the degree of pluripotency of ES cells, the c-Myc/Max complex contributes to the viability and stemness of ES cells [30–32]. Note that while red arrows indicate the activation/stimulation, blue ones show the inhibition/suppression

Given that Myc family transcriptional factors are associated with Max when binding to E-box (enhancer box), one of the DNA response elements [5, 6], mouse ES cells without *Max* gene have been established and investigated to better understand the physiological functions of the c-Myc/Max complex in undifferentiated cells [30]. Unlike *c-Myc*/*N-Myc* double-knockout (DKO) ES cells, the depletion of *Max* gene is accompanied by the loss of the undifferentiated state in ES cells through the activation of mitogen-activated protein kinase (MAPK) signal pathway. The expression levels of Sox2, Oct3/4 and Nanog gradually decrease upon the loss of *Max* gene expression. In contrast, the absence of *Max* gene expression results in the up-regulation of endoderm markers (*Gata4*, *Gata6*, and *Sox17*), ectoderm markers (*Fgf5*, *Nestin*, *Pax6* and *Sox1*), and a trophectoderm marker (*Cdx2*) [30]. The transcription amount of *c-Myc* gene are drastically increased by LIF-mediated Janus kinase (JAK)/STAT3 signal pathway, while the c-Myc protein is stabilized by phosphorylated extracellular signal-regulated kinase (ERK) [31] (Fig. 1). Although *Max*-deficient ES cells undergo extensive apoptotic cell death with caspase-3 activation, *c-Myc*/*N-Myc* DKO ES cells are viable, but these DKO cells fail to maintain the pluripotent capacity. Notably, the c-Myc/Max complex enhances the self-renewal potential of pluripotent ES cells by inhibiting MAPK signaling which is activated by LIF [30]. The c-Myc/Max complex also directly inhibits the expression of *Gata6* gene via miR17-92 cluster, which in turn prevents the ectopic differentiation both in ES and iPS cells [31, 32] (Fig. 1). Furthermore, DKO or the pharmacological inhibition of Myc activity robustly reduces transcription, splicing, and protein synthesis, which is responsible for the proliferation arrest of ES cells. Signal pathways associated with survival and maintenance of ES cells such as interleukin-6 (IL-6) signaling are enriched in DKO ES cells [33]. DKO ES cells display an increased level of cellular adhesion and processes associated with maintenance and survival and enter a state of biosynthetic quiescence, which is characterized by a strong reduction of protein and nucleic acid synthesis.

From the perspective of the relationship with Wnt signal pathway and Polycomb complex, BMI1, one of the polycomb group proteins, has been shown to activate the canonical Wnt signal pathway by repressing the expression level of DKK family members, which leads to the up-regulation of c-Myc [34, 35]. c-Myc in turn up-regulates BMI1 via a c-Myc binding E-box site which is present in its promoter. BMI1 can also directly activate expression of certain Wnt factors, resulting in up-regulation of c-Myc and ultimately transcriptional up-regulation of BMI1 [35]. In addition, the ectopic activation of Myc reveals a positive feedback loop by repressing the Wnt antagonists via polycomb repressive complex 2 (PRC2) recruitment. Myc plays an central role in establishing an epigenetic memory in ES cells by sustaining the self-reinforcing regulatory transcription networks mediated by the potentiation of the Wnt/β-catenin signal transduction and the inhibition of autocrine FGF4/ERK pathway, thereby recapitulating the ground state of ES cells [34–36].

**Fig. 2:**
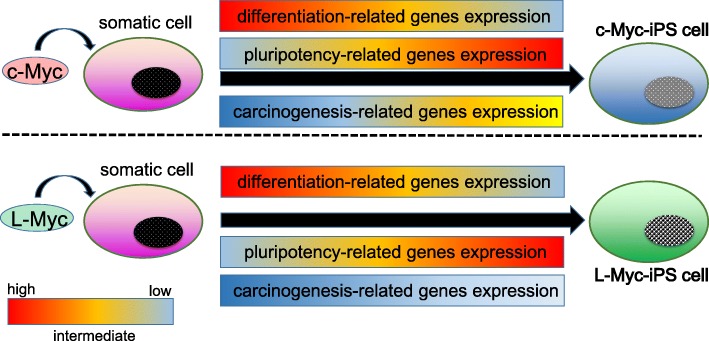
The difference between c-Myc-induced and L-Myc-induced iPS cells. The conventional type of iPS cells established by c-Myc transfection significantly decrease the expression level of differentiation-related genes. Instead, they can acquire stemness, which is defined by self-renewal and multi-lineage differentiation potentials, by the drastic up-regulation of pluripotency-related genetic expression. By striking contrast, the major role of the transcriptional factor L-Myc to generate iPS cells highly depends on the suppression of the genes which might be involved in tumorigenicity as well as the differentiation-related genes, thereby ruling out the possibility of malignant transformation [41].

Recently, there has been a growing interest in the novel function of Myc family in stem cells owing to the increasing number of researchers trying to reveal the molecular machinery of c-Myc and N-Myc in the generation of iPS cells [28, 37, 38]. iPS cells were originally generated using murine fibroblasts by the retroviral introduction of four transcription factors; Sox2, Oct3/4, Klf4, and c-Myc [37]. Mouse iPS cells are indistinguishable from ES cells in morphology, proliferation and gene expression pattern [39]. Selection of iPS cells depending on Nanog expression level (Nanog iPS cells) induces the germline-competent iPS cells with increased genetic expression and DNA methylation pattern closely similar to ES cells compared with iPS cells enriched by Fbx15 expression. Remarkably, the four transgenes compose of Oct3/4, Sox2, c-Myc and Klf4 are strongly silenced in Nanog iPS cells [38]. More than one-half of conventional iPS cells-derived tumors develop within 1 year after birth of chimeric mice. Reactivation of the c-Myc has been detected in these tumors [38, 40, 41]. By striking contrast, chimeric mice derived from the c-Myc-deficient iPS cells do not exhibit an increased incidence of tumor formation [42]. The efficiency of iPS cells generation is significantly declined without c-Myc transfection. Indeed, c-Myc is used in most of the reported experimental methods to generate iPS cells without viral integration [43–46]. Importantly enough, the significance of L-Myc protein to establish iPS cells efficiently without tumor formation capacity has recently attracted much attention since 2010 [41]. L-Myc is composed of the shorter amino acid sequences in the N-terminal region than the other two members of Myc family, which is consistent with significantly reduced transformation activity in the cultured cells [47–49]. In spite of the weak transformation activity of L-Myc, this Myc protein has been found to exhibit a stronger and more specific activity in promoting iPS cells generation. Furthermore, the ability of germline transmission of L-Myc is similar to that of c-Myc [41]. Taken together, while c-Myc functions as a “double-edged sword,” promoting both iPS cells generation and malignant transformation, L-Myc contributes to the nuclear reprogramming with the suppression of differentiation-associated genes expression (Fig. 2).

## Emerging roles of Myc in terms of the carcinogensis of the difficult-to-treat tumors

c-Myc, which is located at chromosome 8q24, is one of the proto-oncogenic genes which is most frequently involved in human carcinogenesis. The *c-Myc* gene was initially identified as the homolog of the v-myc oncogene in avian acute leukemia virus around 40 years ago [50]. Direct evidence of involvement of c-Myc in human cancer cells came from the discovery and identification of the c-MYC gene at 8q24 and its translocation onto the immunoglobulin heavy chain locus in human Burkitt lymphoma [9, 10]. It is sure that c-MYC regulates various cancer cellular functions, including cell cycle, cellular survival, proliferation, metabolic reprogramming [2, 3, 10, 12]. Notably, c-Myc has been shown to induce the dedifferentiation towards a progenitor-like state mediated by the significant down-regulation of lineage-specifying transcriptional factors, which results in the inhibition of luminal-specific enhancers such as histone H3 lysine 9 monomethylation (H3K4me1) and histone H3 lysine 27 acetylation (H3K27ac) [51]. Indeed, c-Myc binding achieves a peak at the center of the H3K27ac-enriched region among c-Myc-targeted *de novo* enhancers, which strongly suggests a direct contribution to the deposition of this active histone mark. The down-regulation of GATA3 and ESR1, both of which are master regulators of mammary gland morphogenesis and luminal cell differentiation, is mainly regulated by c-Myc which binds to their *cis*-regulatory elements [51, 52]. Thus, c-Myc-induced oncogenic and epigenetic reprogramming leads to the acquisition of cancer stem-like cells (CSCs)-associated properties and induction of the intra-tumoral heterogeneity. It is widely accepted, however, that N-Myc plays a central role in therapeutic-resistant neuroendocrine tumors including specific type of lung cancers, medulloblastoma and neuroblastoma [53–55]. Given the relatively ignorance of the detailed function of N-Myc compared with c-Myc in difficult-to-cure cancers, the molecular function and machinery of N-Myc in the neuroendocrine tumors are mainly described in this section.

N-Myc is overexpressed both in tumors of the nervous system such as neuroblastoma, medulloblastoma, retinoblastoma, astrocytoma, and glioblastoma multiforme, and in non-neuronal cancer characterized by castration-resistant neuroendocrine-typed prostate cancer, hematological malignancies, rhabdomyosarcoma, Wilms tumor, small-cell lung cancer, and pancreatic tumor [55, 56]. In many clinical cases, c-Myc- or N-Myc-driven tumors are likely to arise from the cell lineages which express c-Myc or N-Myc during the normal development of each tissue. However, the multiple functions of N-Myc in tumor cells differ from those of c-Myc within a specific cell lineage. For a typical example, N-Myc is expressed in self-renewing, quiescent stem cells, but this expression switches to c-Myc upon differentiation to transit-amplifying progenitors in the hematopoietic lineage [57]. This fact strongly suggests that N-Myc plays an essential role in the activation of stem cell-like properties defined by both self-renewal and multi-differential potential. In the case of prostate cancer, a growing body of evidence suggests that N-Myc plays a crucial role in the lineage switching from an epithelial origin to a more neuroendocrine one. A shift in expression among Myc family during tumor progression can be associated with a shift in cellular lineage, tumor progression, and treatment resistance [55, 57]. In terms of the epigenetic regulation, N-Myc binds to the enhancers of the gene encoding androgen receptor (AR) and forms a complex with the nuclear receptor which is dependent on its interaction with enhancer of zeste homolog 2 (EZH2) [58]. Furthermore, the catalytic activity of EZH2 increases the number of the complex composed of N-Myc, AR, and EZH2-PRC2 [58, 59]. Enhanced levels of EZH2 protein expression and EZH2 catalytic activity play a crucial role both in murine models overexpressing N-Myc and in human castration-resistant prostate cancer cells. N-Myc redirects EZH2 activity to N-Myc target gene promoters, resulting in the transcriptional suppression, whereas EZH2 inhibition reverses N-Myc-driven genetic regulation. Importantly, N-Myc sensitizes tumor cells to EZH2 inhibitors both in vitro and in vivo [58].

N-Myc has been reported to be amplified in 15-20% of small-cell lung cancer (SCLC) tissues [60, 61] and associated with poor clinical prognosis and therapeutic response to chemotherapy [62, 63]. N-Myc amplification rarely occurs in other type of pulmonary pathohistological cancers including adenocarcinoma and squamous cell carcinoma. N-Myc amplification occurs in approximately 40% of neuroendocrine prostate cancer type, which is commonly recognized concurrently with the genetic amplification of the aurora kinase A [64, 65]. N-Myc amplification is likely to occur early before the development of metastasis in both small-cell lung and prostate cancers. N-Myc amplification is frequently observed in medulloblastoma and N-Myc overexpression is positively correlated with poor clinical outcomes [66]. N-Myc is implicated as a critical driver of tumor initiation and progression in the preclinical models of both SCLC and medulloblastoma [55]. In genetically engineered mouse models (GEMMs), it has been shown that murine N-Myc-driven SCLC expresses high level of NEUROD1, which is a key transcriptional factor for the survival and proliferation of neuroendocrine tumor cells [55, 67]. Based on *in situ* immunostaining patterns for achaete-scute homolog 1 (ASCL1) and NEUROD1, it is proposed that N-Myc-driven cancer cells emerge among ASCL1-positive precursor cells, and these early-staged cancer cells initially exhibit classic morphology. With the passage of time, it seems likely that tumor cells change into an ASCL1 (low)/NEUROD1 (high) expression pattern which is coincident with the appearance of variant morphology phenotype in GEMMs [67]. Because the overexpression of NEUROD1 has been linked to the development of metastases and aggressive SCLC phenotypes [68], it has been suggested that N-Myc activation results in the variant characteristics via NEUROD1 signal activation. From the therapeutic perspectives, N-Myc expression levels, the neuroendocrine-low expression profile, and variant pathohistopathology are all expected to serve as the useful biomarkers to predict the sensitivity to Aurora kinase inhibition in the clinical settings. It has been shown that Aurora kinase inhibition is highly likely to improve the chemotherapy response in vivo, which strongly suggests that the patients with N-Myc-amplified SCLCs exhibit the significant clinical benefit from the first-line therapy with Aurora kinase inhibitors in combination with the conventional chemotherapy [67, 69, 70]. In addition, it has been very recently shown that α subunit of the epithelial sodium channel (αENaC) is a downstream therapeutic target molecule of ASCL1-positive in pulmonary neuroendocrine tumor [71–73]. Amiloride has been shown to decrease the proliferation of neuroendocrine lung cancer cells which highly express ASCL1 but not in cancer cells with low ASCL1 expression. Amiloride, which is an oral potassium-sparing diuretic agent, has been reported to have anti-tumor and anti-metastatic functions both in vitro and in vivo, which is the typical example of the drug-repositioning (DR) targeting αENaC [73, 74]. Conventional drugs are not only pharmacologically safe but also less expensive than specialized anti-cancer agents. DR strategy leads to the better understanding of the molecular machineries of how the conventional drugs show anti-tumor effects [74]. Table 1 shows the promising drugs of the current DR targeting Myc family. For a typical instance, it has been demonstrated that valproic acid, which has been used for the treatment of depression and epilepsy such as tonic-clonic seizures, contributes to the up-regulation of CDKN1A/B (p21/CIP1/WAF1, p27/KIP1) and down-regulation of c-Myc, thereby augmenting mammalian target of rapamycin (mTOR) inhibitor to induce autophagic cell death in cutaneous T cell and Burkitt lymphomas [74–76].

**Table 1:** Typical examples of drug re-positioning targeting Myc in tumor cells

Name of the agent	Conventional applications	Mechanisms of action	References
Valproic acid (a short-chain fatty acid HDAC inhibitor)	Migraine attacks, depression and epilepsy such as tonic-clonic seizures	To upregulate CDKN1A/B (p21, p27) and downregulate c-Myc, thereby inducing autophagic cell death in cutaneous T cell lymphoma and Burkitt lymphoma	[75, 76]
Retinoic acid (an analogue of vitamin A)	Acne vulgaris, psoriasis, and AML M3 (acute promylocytic leukemia; APL)	To downregulate N-Myc in cooperation with interferon-γ (IFN-γ), thereby inducing differentiation in neuroblastoma	[85, 232, 233]
Bortezomib (proteasome inhibitor)	Multiple myeloma	To up-regulate the proapoptotic protein NOXA in malignant melanoma and Burkitt lymphoma directly dependent on c-MYC	[234, 235]
Sulfasalazine (cystine/glutamate antiporter inhibitor)	Ulcerative colitis and rheumatoid arthritis	To disrupt the circadian rhythm of transferrin receptor 1 gene expression regulated by c-Myc and to decrease glutathione synthesis by the inhibition of cystine uptake via system Xc(-)	[117, 130, 236]

Neuroblastoma is the most common solid endocrine tumor in pediatric patients and the third most common pediatric tumor overall. It most commonly occurs in the adrenal medulla, which secretes epinephrine (also referred to as adrenaline), norepinephrine (also known as noradrenaline), and a small amount of dopamine in response to the physiological stimulation by the sympathetic preganglionic neurons [77, 78]. Neural crest cells have been demonstrated to be the cell-of-origin of neuroblastoma, which undergo the multi-lineage differentiation [79]. Surprisingly, more than 95% cases of neuroblastoma have wild-type p53 [80]. Amplified N-Myc directly binds with the tetrameric form of p53 at the C-terminal domain in this neuroendocrine tumor. N-Myc and p53 exhibit the co-localization in the nucleus and alter p53-dependent transcriptional responses that are necessary for DNA repair, anti-apoptosis, and lipid metabolic reprogramming [81].

**Fig. 3:**
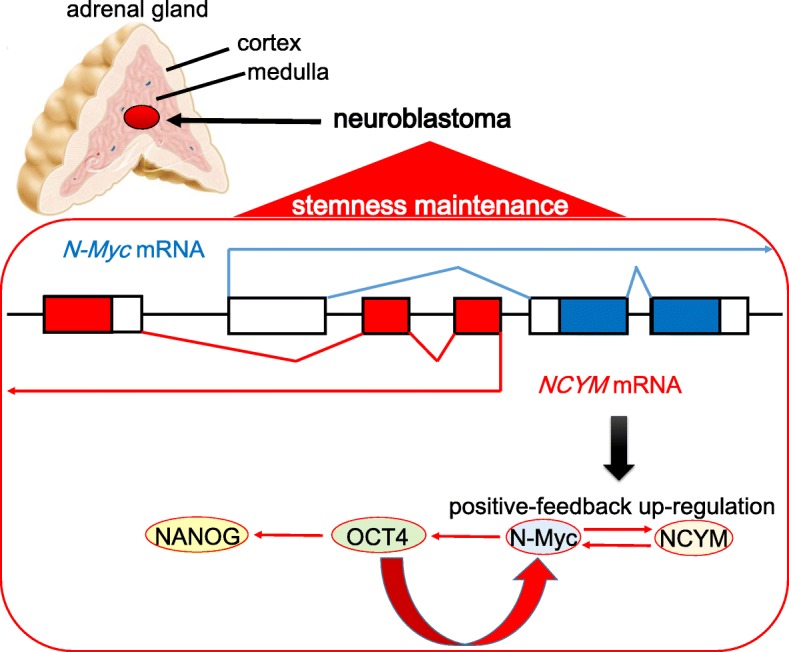
The positive-feedback machinery of N-Myc to induce and maintain the stemness of neuroblastoma. Neuroblastoma is the most common high grade endocrine childhood-onset tumor, which often occurs in the adrenal medulla and sympathetic ganglions. N-Myc exhibits the significant up-regulation in cooperation with NCYM, which is a *cis*-antisense transcript of oncogene product N-Myc and a functional long non-coding RNA. N-Myc enhances the expression level of Oct4, which play a pivotal role in the maintenance of undifferentiated conditions of medulloblastoma. Oct4 has dual functions; to induce the expression of Nanog, another key transcriptional factor, and to enhance N-Myc expression in a positive-feedback manner [86]. While the filled areas in blue or red indicate opening reading frames (ORFs), the blank areas mean the exons encoding prime untranslated regions such as 3’ and 5’ UTR

It is remarkable that some neuroblastoma cells continue to exhibit the stemness characterized by self-renewal and multipotent potentials and highly express several genes related to CSCs, such as N-Myc, Oct4 and LIN28 [82, 83]. Intermediate-type neuroblastoma cells tend to express high level of Oct4 and differentiate into neuroblastic-type or substrate adherent-type tumor cells in response to the retinoic acid [84]. Notably, neuroblastoma cells positive for both Oct4 and Tenascin C have been reported to function as the progenitor cells of endothelial cells of the difficult-to-treat childhood-onset neuroendocrine tumor, thereby promoting the neovascularization of the tumor microenvironment [82]. Furthermore, Oct4 is expressed in the side-population of the neuroblastoma tumor cells [85]. In spite of these close correlations between Oct4 expression and the cancer stem cell-like characteristics of the neuroblastoma, the functional roles of N-Myc in neuroblastoma pathogenesis remain unknown in details. It has been recently revealed that the expression level of Oct4 is associated with unfavorable clinical prognosis and therapeutic responses in N-Myc-amplified neuroblastomas, but not in N-Myc-non-amplified tumors [86]. N-Myc expression exhibits the inverse correlation with c-MYC in neuroblastomas and that the low transcriptional level of Klf4 is related with the poor clinical outcome of neuroblastoma patients [87, 88]. NCYM is a *cis*-antisense transcript of oncogene product N-Myc, which has been considered to be a long non-coding RNA. It has been recently reported that NCYM is a newly evolved coding *de-novo* gene which is conserved only in a taxonomically-restricted group including humans and monkeys [89–91]. Importantly, NCYM is co-amplified with MYCN in human neuroblastomas. It has been shown that NCYM is positively correlated with Nanog expression and is inversely correlated with both Klf4 and c-MYC [86]. As the overexpression of Oct4 induces aberrant transcription levels of Nanog, the correlation between Nanog, N-Myc and NCYM in neuroblastoma tumor cells can be explained by Oct4, which is their common upstream regulatory molecule. Thus, NCYM protein stabilizes N-Myc, resulting in the stimulation of Oct4 expression, whereas Oct4 induces both N-Myc and NCYM via direct transcriptional activation of N-Myc (Fig. 3). Collectively, there is a positive feedback machinery composed of N-Myc, NCYM, and Oct4, leading to the maintenance of the high expression levels and enhanced self-renewal ability of undifferentiated cells in N-Myc-amplified neuroblastoma tissues. Therefore, the differentiation-inducing therapy by retinoic acid treatment improves the overall survival of the patients with N-Myc-amplified neuroblastomas, and all-*trans* retinoic acid (ATRA) treatment abrogated the mutual transcriptional regulations between N-Myc, NCYM, and Oct4, all of which induce the differentiation of neuroblastoma precursor cells [86, 92, 93]. Notably, the same holds true for the treatment of AML M3 (acute promyelocytic leukemia; APL) [94, 95] (Table 1).

## Pathophysiological significance of Myc expression in terms of cancer metabolic reprogramming

**Fig. 4:**
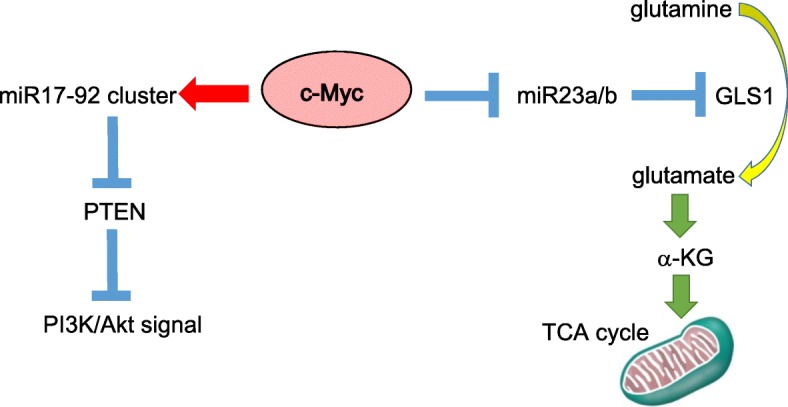
The interaction between oncogenic c-Myc and several microRNAs. c-Myc plays a central role in the metabolic reprogramming through the interaction with microRNAs such as miR17-92 cluster, miR23a/b, and miR34a. The association of c-Myc with miR17-92 cluster inhibits phosphatase and tensin homologue deleted on chromosome 10 (PTEN), thereby augmenting the PI3K-Akt-mammalian target of rapamycin (mTOR) axis [112–114]. In addition, the interaction of c-Myc with miR23a/b promotes the conversion of glutamine into glutamate with the up-regulation of glutaminase 1 (GLS1) [109–111]. Note that while the red arrow indicates “activation,” blue bars indicate “suppression”

Accumulating evidence strongly suggests that oncogenic levels of Myc expression results in the metabolic reprogramming specific to cancer cells [96–100]. c-Myc overexpression leads to the “glutamine addiction” for the maintenance of the integrity in mitochondrial TCA cycle (also referred to as Krebs cycle) [101]. Previous studies indicated that lactate dehydrogenase (LDH) A isoform induction by c-Myc is required for Myc-transformation [102–104], which is responsible for the diversion of glucose-derived pyruvate into lactate. In spite of this fact, Myc-transformed cancer cells exhibit an increased mitochondrial mass and increased rate of oxygen consumption [105, 106]. Furthermore, it has been reported that Myc-overexpressing tumor cells are exquisitely sensitive to the inhibitor of the mitochondrial electron transport chain [107, 108]. This paradoxical phenomenon can be explained by the accumulation of glutamine, the major catabolizing bio-energetic substrates in mitochondrial TCA cycle [109]. Myc-induced transformation leads to the conversion from glucose to glutamine as the oxidizable substrate which is essential to maintain TCA cycle activity. c-Myc binds to the promoters and induces the expression of several crucial regulatory genes which are involved in glutaminolytic metabolism. It has been demonstrated that supra-physiological levels of Myc expression associated with oncogenic transformation are both necessary and sufficient for the induction of glutaminolysis to the excessive level that results in “glutamine addiction” specific to tumor cells [109]. Interestingly enough, c-Myc directly binds to the transcription subunit of microRNA (miRNA)-23a/b and subsequently contributes to the up-regulation of mitochondrial glutaminase 1 (GLS1) via the induction of ASCT2/SLC1A5 transporter [109–111]. Moreover, the association of c-Myc with miR17-92 cluster has been shown to inhibit the activity of phosphatase and tensin homologue deleted on chromosome 10 (PTEN), which is why this miRNA cluster activates PI3K-Akt-mTOR axis [112–114]. That is why the complex crosstalk between miRNA and Myc is considered to be partially responsible for metabolic reprogramming (Fig. 4).

Indeed, some, but not all, Myc-induced transformed cancer cells tend to be dependent on glutamine metabolism [115]. For a typical instance, triple-negative breast cancer (TNBC) lacking in the expression of estrogen receptor, progesterone receptor, and the tyrosine kinase receptor HER2/EGFR2, exhibits the significant dependence on the glutamine metabolism due to the coordination between the amino acid transporters such as xCT/SLC7A11 and ASCT2/SLC1A5 [100, 116]. xCT/SLC7A11 transporter uptakes cystine in exchange for glutamine, which is essential for reduced form of glutathione (GSH) synthesis to reduce reactive oxygen species (ROS) in the tumor microenvironment [74, 117–120], while ASCT2/SLC1A5 transporter uptakes glutamine in a collaborative manner with xCT/SLC7A11 [101, 121]. Glutamine is simultaneously imported mediated by ASCT2/SLC1A5 transporter and exported in exchange for leucine mediated by LAT1/4F2 (CD98 heavy chain) antiporter [116, 122]. The glutamine uptake promotes the synthesis of α-ketoglutarate (α-KG, also referred to as 2-oxoglutarate), which is the metabolic intermediate of TCA cycle in mitochondria, thereby also facilitating the synthesis of nucleotides required for DNA replication and cellular proliferation [100]. Therefore, the metabolic reprogramming in cancer cells, which is orchestrated by the increased expression and the interaction of amino acid transporters, contributes to the excessive dependence on the glutamine metabolism, and more importantly, this metabolic reprogramming is expected to protect cancer cells from accumulated ROS via the robust GSH synthesis.

In addition, the ability for Myc to induce glutaminolysis in tumor cells does show a potentially beneficial effect with the active production of reduced form of nicotinamide adenine dinucleotide phosphate (NADPH) [101, 123]. It has long been believed that the major substrate origin necessary for NADPH synthesis during cellular growth and proliferation occurs through the oxidative arm of the pentose phosphate shunt [124, 125]. However, recent research indicates that the Myc-induced transformed cells exhibiting the aerobic glycolysis, which is also referred to as the Warburg effect [101, 126, 127], produce the majority of their ribose biosynthesis through the non-oxidative arm of the pentose phosphate shunt [128]. *De novo* nucleotide synthesis with ribose synthesized in the non-oxidative arm of the pentose phosphate shunt is likely to rapidly lead to the intracellular depletion of NADPH in the absence of a compensatory mechanism to generate NADPH. Thus, the ability for Myc to stimulate NADPH production via enhanced glutaminolysis provides the Myc-induced transformed cell with a finely-regulated machinery underlying the synthesis of the enough amount of NADPH necessary for tumor cell proliferation.

**Fig. 5:**
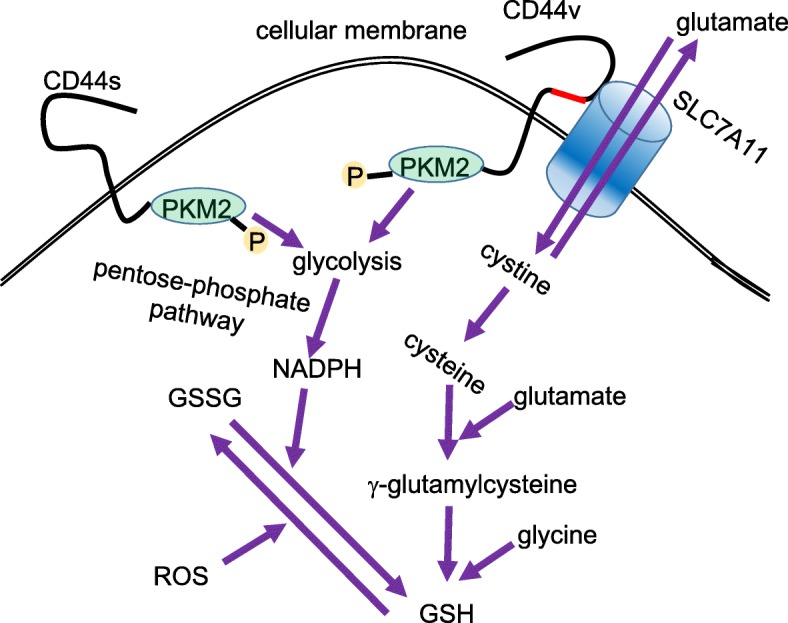
Function of CD44 in promoting resistance to oxidative stress with metabolic reprogramming. Alternative splicing of the CD44 gene results in the generation of multiple protein isoforms. CD44 standard isoform (CD44s) binds to PKM2, thereby promoting aerobic glycolysis in cancer cells (Warburg effect) and the pentose phosphate pathway (PPP). The PPP provides enough NADPH to convert oxidative form of glutathione (GSSG) to reduced form of glutathione (GSH) [147, 152]. On the other hand, CD44 variant isoform (CD44v) is overexpressed in epithelial cancer stem cells (CSCs), and its colocalization with the xCT subunit of system Xc(–), a glutamate/cystine antiporter, promotes the uptake of cystine and the consequent synthesis of the antioxidant GSH, which reduces the amount of reactive oxygen species (ROS) [117, 119, 147]. The red region of CD44v indicates the variable exons 8-10 which are inserted by ESRP1-induced alternative splicing

Remarkably, CD44 variant isoform (CD44v), including sequences encoded by variable exons 8, 9, and 10, interacts with and stabilizes xCT/SLC7A11 transporter at the cellular membrane of CSCs [117–119]. Epithelial splicing regulatory protein 1 (ESRP1), one of the RNA-binding proteins, has been identified as to affect alternative splicing and induce CD44v expression [129, 130]. Chromatin immunoprecipitation (ChIP) sequencing analysis at the *ESRP1* locus has clarified that CD44v-positive tumor cells manifest the enrichment of H3K4me3 at the transcription start site, while CD44v-negative cells exhibit that of H3K27me3. This fact strongly suggests that ESRP1 expression is tightly regulated by the epigenetic modifications of the *ESRP1* locus as well as by the epithelial-mesenchymal-transition (EMT) [130]. As described above, xCT/SLC7A11 transporter, together with CD98 heavy chain (CD98hc), forms an antiporter known as system Xc(–) that exchanges intracellular glutamate for extracellular cystine [117, 131]. Given that cysteine as well as glycine and glutamate are essential substrates for the synthesis of GSH, CD44v promotes GSH synthesis by increasing the import of cystine, thereby enhancing the intracellular concentration of cysteine (Fig. 5). The elimination of ROS by GSH inhibits the activation of p38 MAPK signaling pathway [117, 132], preventing ROS-induced senescence, apoptosis, or ectopic differentiation of cancer stem-like cells. This ESRP1-CD44v-xCT-GSH axis enables CD44v-positive breast CSCs to exhibit the distant metastasis to the lungs despite of the exposure to excessive ROS generated by tumor-entrained neutrophils (TENs) [130, 133]. That is mainly why persistent cancer cells after the exposure to ROS are expected to highly express c-Myc in the minimal residual disease (MRD). Furthermore, ferroptosis, or iron-ion-dependent regulated necrotic cell death, is related to excessive ROS-induced lipid peroxidation [74, 134–136]. Activation of system Xc(–) prevents ferroptosis mediated by glutathione peroxidase 4 [137–139]. Taken together, ESRP1-CD44v-xCT-GSH axis protects CSCs from ROS-induced cellular damage.

In the glycolytic process, pyruvate kinase (PK) catalyzes the last reaction, the transfer of a high-energy phosphate group from phosphoenolpyruvate to ADP, producing ATP and pyruvate. The highly active PK isoenzyme type M1 (PKM1) is expressed in tissues that consistently need high levels of energy, such as skeletal muscle, heart, and brain. By contrast, PKM2 is expressed in most tissue cells except adult muscle, brain, and liver. Moreover, PKM2 is the predominant PK isoform in proliferating cancer cells [140–142]. One of the important characteristics of cancer cells that differentiates them from normal cells is that cancer cells use glycolysis to produce ATP regardless of the local availability of molecular oxygen (the Warburg effect) [126, 127, 143, 144], and PKM2 plays a critical role in this process [141, 145–147]. Paradoxically, it has been recently reported that PKM1 accelerates the glucose catabolism including both glycolysis and TCA cycle, and more importantly, enhances the malignant potential of N-Myc-driven neuroendocrine tumors such as SCLC via efficient mitophagy, the selective autophagy-dependent degradation of old and dysfunctional mitochondria generating cytotoxic ROS [142]. Importantly, high levels of c-Myc activity are responsible for the enhanced PKM2/PKM1 ratios [148, 149]. Given that c-Myc also promotes glycolysis with the up-regulation of glucose transporter type 1 (GLUT1), hexokinase 2 (HK2) and pyruvate dehydrogenase kinase 1 (PDK1) in collaboration with hypoxia-inducible transcription factors (HIFs), particularly HIF-1 [97, 150, 151], c-Myc promotes the adaptation to the hypoxic microenvironment. By striking contrast, low PKM2 activity inhibits the conversion of pyruvate to lactate, thereby promoting the flow of glycolytic intermediates into biosynthesis for the generation of NADPH [147, 152]. Expression of CD44 contributes to the defense against ROS through two different mechanisms. Firstly, CD44-intracellular domain (ICD) of CD44 standard isoform (CD44s) interacts with and suppresses PKM2 activity by increasing its phosphorylation, thereby promoting the glycolytic pathway and leading to the antioxidant status (increased GSH and reduced ROS) of CSCs [119, 147]. Secondly, CD44v isoform interacts with and stabilizes xCT/SLC7A11 transporter, an essential component of the cystine-glutamate transporting system Xc(–), thereby promoting cystine uptake for GSH synthesis [117, 119, 153]. Collectively, both CD44s and CD44v isoforms protect CSCs, which are often exposed to high levels of ROS in the tumor microenvironment (Fig. 5).

In terms of the lipid metabolic reprogramming, it has been revealed that c-Myc-dependent metabolic dysregulation is crucial for the survival and proliferation of c-Myc-overexpressing TNBC. A lipid metabolism gene signature tends to be enriched in patients with TNBC according to TCGA [154, 155]. It is notable that the interaction between wild-type p53 and amplified N-Myc causes the lipid metabolic reprogramming [81]. The degree of AMP-activated protein kinase (AMPK) signal activation exhibits the inversed relationship with c-Myc [156, 157]. AMPK-mediated phosphorylation of the palmitate cell surface receptor CD36 has also been reported to increase its rate of cycling between the cell membrane and intracellular compartments, thereby influencing on the normal balance between fatty acid β-oxidation (FAO) and the accumulation of the cytoplasmic lipid droplets. Besides, CD36 has been recently identified as to contribute to the activation of mitochondrial FAO, leading to the enhanced metastasis to the lymph nodes [156–158]. Interestingly enough, it has been shown that N-Myc is highly expressed in CSCs of hepatocellular carcinoma (HCC) which depends on canonical Wnt/β-catenin signal pathway [159, 160]. Lipid biogenesis has specifically been demonstrated to be essential for the proliferation of N-Myc-derived tumors, which explains why the inhibitors of fatty acid synthesis show the specific toxicity to tumor cells highly expressing N-Myc [161]. Both the restricted expression of N-Myc in CSCs of HCC and the susceptibility to acyclic retinoid (ACR), one of the derivate chemicals of vitamin A, hold much promise in the novel therapeutic strategy to prevent the recurrence of *de novo* HCC [159]. Given the decreased subpopulation of CSCs of HCC highly expressing both EpCAM and N-Myc after the treatment with ACR, ACR is expected to induce the differentiation of CSCs with N-Myc down-regulation [160].

It has been very recently reported that c-Myc contributes to the metabolic reprogramming of pyrimidine synthesis mediated by the up-regulation of *CAD* gene encoding carbamoyl-phosphate synthetase 2, *UMPS* gene encoding uridine monophosphate synthetase, and *CTPS* gene encoding cytidine triphosphate synthase in colon adenocarcinoma cells [162]. Knockdown of pyrimidine synthesis genes mainly regulated by c-Myc results in the suppression of the proliferation of the colorectal tumor cells, which is quite similar to *c-Myc* knockdown. In contrast, the knockdown of metabolic enzymes-coding genes necessary for purine synthesis does not affect the proliferation of colorectal cancer cells [162–164]. As many as 231 genes have been identified in a total of more than 300 metabolic reactions, including the pentose phosphate pathway, purine/pyrimidine synthesis pathway, fatty acid oxidation pathway and MAPK signaling pathway [162]. Among of these various pathways, almost all metabolic genes of the *de novo* purine/pyrimidine synthesis pathway are significantly up-regulated in colon cancer cells, which are positively correlated with the enhanced expression level of c-Myc [162, 165]. Therefore, pyrimidine synthesis pathway can be a potential target for novel therapeutic target of colorectal tumor highly expressing c-Myc. It is highly likely that this novel finding has implications for future therapeutic approaches targeting c-Myc-regulated metabolic reprogramming for colorectal cancer patients.

Given the various kinds of metabolic reprogramming in tumor cells, it seems to be quite important to describe the therapeutic strategy how to overcome the difficult-to-treat tumors in terms of the metabolic shift driven by Myc family. Glutaminolysis is the major metabolic reprogramming triggered by c-Myc in cancer cells [101, 166–168]. Therefore, the identification of the novel specific inhibitors against glutaminase (GLS) has recently become a field of intensive research and then a variety of small molecule inhibitors have been developed. As of this writing, the best characterized GLS inhibitor is bis-2-[5–phenylacetamido-1, 2, 4-thiadiazol-2-yl] ethyl sulfide (BPTES). BPTES inhibits the dimer-to-tetramer transition of GLS in the allosteric manner, which is essential for the activation of the enzyme [169]. A large number of derivatives of BPTES such as CB-839 have been designed [170–172]. BPTES effectively inhibits the proliferation of numerous types of malignancies, including c-Myc-dependent hepatocellular carcinoma, malignant lymphoma and renal cell carcinoma [172].

## The disruption of degradation pathway of c-Myc in cancer cells leading to therapeutic resistance

The abundance of numerous intracellular proteins, which are involved in various cellular physiological and pathological processes including cell cycle progression, cellular proliferation, and apoptotic cell death, is regulated by the ubiquitin proteasome system (UPS) through ubiquitination-mediated degradation by the 26S proteasome [173–175]. Elevated degradation of specific kinds of tumor suppressor gene products or impaired destruction of oncogenic proteins appears to bring about tumor development due to the mutated components of the UPS [174–176]. It is widely accepted that UPS is composed of the three different molecules; ubiquitin-activating enzyme (E1), the ubiquitin-conjugating enzyme (E2), and ubiquitin-protein ligases (E3). The E1 utilizes ATP to activate ubiquitin for conjugation and transfers it to E2. The E2 enzyme interacts with a specific E3 ligase and transfers the ubiquitin to the target protein which is the substrate for UPS-dependent degradation [173, 174, 177].

**Fig. 6:**
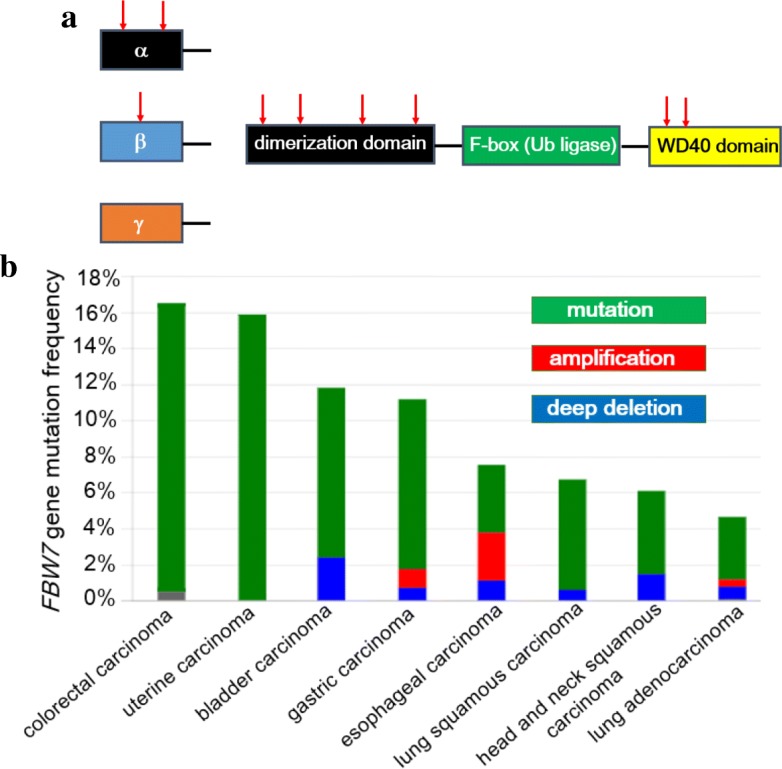
Scheme of FBW7 structure and the frequency of genetic mutations in gastric cancer patients. **a** All of the three isoforms of FBW7 are different each other at the N-terminal domain but contain evolutionarily well-conserved interaction domains in the C-terminus (F-box ubiquitin ligase domain and WD40 repeated substrate binding domain). The previous investigation reveals that 7 out of 80 gastric cancer patients (8.8%) had missense mutations in *FBW7* gene [191]. Note that red arrows indicate missense mutations. Given the space available, repeated WD40 domains are abbreviated in this Figure. **b** The Cancer Genome Atlas (TCGA) information provided by the cBio Cancer Genomics Portal at Memorial Sloane Kettering Cancer Center (http://www.cbioportal.org/) shows that more than 15% of patients of both uterine carcinoma and colorectal adenocarcinoma harbor the mutation of *FBW7* gene

The SCF (SKP1-CUL1-F-box protein) E3 ligase complex, which consists of Skp1 (S-phase kinase-associated protein 1), Cul1, Rbx1/Roc1, and a variable subunit denoted as the F-box protein, has been well investigated among E3 enzymes [178–181]. Of note, F-box protein determines the specificity of the UPS substrate via binding of the target proteins for ubiquitination and degradation. So far, as many as 70 kinds of putative F-box proteins have been identified in human genome, although the function and their substrates of most F-box proteins still remain unknown in details [177, 182, 183]. One of the well-investigated F-box-containing proteins is F-box and WD repeat domain-containing 7 (FBW7), also known as FBXW7 [183–185]. About 40 years ago, the first member of the *FBW7* gene family was originally identified in budding yeast and named as cell division control protein 4 (Cdc4) [186]. It has been revealed that human *FBW7* gene is located on chromosome 4 and encodes three different transcripts (isoforms α, β and γ) derived from the identical gene locus by the alternative splicing [187–189]. All three isoforms are different each other at the N-terminal domain but contain evolutionarily well-conserved interaction domains in the C-terminus (F-box and WD40 repeats) (Fig. 6a). Three FBW7 α, β and γ isoforms are localized in the nucleoplasm, cytoplasm and nucleolus, respectively [183, 187]. The F-box motif is composed of 40 amino acid residues within each F-box protein which recruits the SCF complex by the direct interaction with Skp1 to form a functional E3 ligase complex [183]. In addition, as many as eight repeats of WD40 which bind to phosphorylated substrates exist at the C-terminal region of FBW7 [190]. According to the precious report, 7 out of 80 gastric cancer patients (8.8%) had missense mutations in *FBW7* gene [191]. It is noteworthy that there existed no mutations in F-box motif. (Fig. 6a). Moreover, uterine carcinoma and colorectal adenocarcinoma show the relatively high frequency of the genetic mutations of *FBW7* locus (approximately 16%) among numerous kinds of solid tumors according to TCGA database provided by the cBio Cancer Genomics Portal at Memorial Sloane Kettering Cancer Center (http://www.cbioportal.org/) (Fig. 6b). Unlike the previous report [191], TCGA analysis shows not only genetic mutations but also amplification and deep deletion in gastric cancer patients. In addition, there have been reported the amplification of *FBW7* gene among the patients with esophageal cancer, gastric cancer, and lung adenocarcinoma (Fig. 6b).

Accumulating evidence strongly suggests that FBW7 serves as a tumor suppressor molecule with the negative regulation of various proteins highly expressed in tumor cells characterized by c-Myc, Notch, Cyclin E and c-Jun [179, 181, 183, 192]. It has been demonstrated that mice with T-cell lineage-specific inactivation of FBW7 are predisposed to the development of the thymic lymphoma [193]. Furthermore, the thymus in these GEMMs contains a uniform population of immature lymphoid cells with necrosis. The presence of thymic lymphoma cells which are positive for both CD4 and CD8 strongly suggests the accumulation of immature T cells in the lymphomas [193]. Mechanistically, the deletion of *FBW7* in T cells develops thymic lymphoma partially due to the excessive accumulation of oncogenic c-Myc. More importantly, the double mutant mice which do not express neither FBW7 nor p53 in T cells develop thymic lymphomas at a markedly increased frequency and with a reduced latency [193], which indicates the potential synergistic interaction between loss of FBW7 and p53 tumor suppressors in facilitating tumorigenesis. Therefore, Myc-dependent T cell lymphomagenesis is accelerated by the decreased level of Myc-induced apoptosis, which is caused by the disruption of p53 function.

To investigate the pathophysiological function of FBW7 in hematopoietic stem cells (HSCs) in the bone marrow (BM), BM-specific *FBW7* knockout mice have been generated and analyzed [194]. More than 50% of mice deficient in FBW7 expression in BM develop T-cell acute lymphoblastic leukemia (T-ALL) within 16 weeks after birth. Lymphoid blasts exhibit the aggressive invasion into many organs including liver, spleen, thymus, and kidneys in the leukemic model mice [194]. Mechanistically, leukemic cells in FBW7-deficient mice fail to exhibit the FBW7-dependent degradation of Notch-1 and c-Myc, which is why the high expression level of Notch-1 and c-Myc in FBW7-deficient BM cells is regarded as responsible for T-ALL development [194, 195]. Intriguingly enough, the cyclin-dependent kinase inhibitor p57, which is highly expressed in HSCs, has been shown to be important for the maintenance of dormant HSCs localized in the niche. Depletion of p57 in HSCs induces the aberrant cellular proliferation in BM and consequently leads to the exhaustion of HSCs population [196–198].

FBW7-deficient mouse embryonic fibroblasts (MEFs) have been established to perform the more precise molecular analysis [199]. It has been reported that FBW7-deficient MEFs detached spontaneously from the culture dish under the conventional culture conditions [199, 200], which suggests the possibility that FBW7 depletion causes anoikis resistance [201]. Unexpectedly, the speed of cellular proliferation of FBW7-deficient MEFs is significantly decreased compared with wild-type control MEFs [199]. Ablation of *FBW7* gene reduces cellular growth mediated by the induction of cell cycle arrest at G_0_/G_1_ phase and the increased frequency of apoptosis [179, 199]. Surprisingly, the inhibition of cell growth by loss of FBW7 in MEFs is accompanied by increased abundance of Notch-1. Furthermore, both induction of cell cycle arrest and increased apoptosis in FBW7-deficient MEFs require Notch-RBP-J (recombination signal binding protein for immunoglobulin κ J region) signal pathway [199, 202]. Cell cycle arrest due to the depletion of FBW7 in MEFs is largely dependent on the p53 pathway, whereas increased apoptosis in these MEFs is mediated in a p53-independent manner [199]. It has been recently demonstrated that the ablation of FBW7 leads to the dysregulated activation of Notch-1, which in turn inhibits normal expression level of p27 and p57 but instead promotes the expression amount of p21 and p53 [203–205]. The expression level of p19 is dependent on c-Myc, while p16 accumulation has been found to be independent on Notch and c-Myc in FBW7-deficient MEFs [179]. Collectively, these unexpected and complicated events suggest that FBW7 may not only play a tumor suppressor role in MEFs, but FBW7 may also have the different effects in different tissues in a context-dependent manner.

**Fig. 7:**
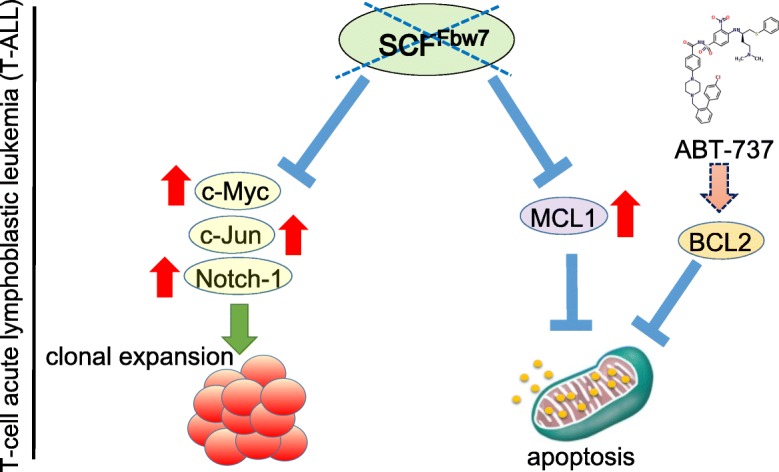
Loss-of-functional mutation in *FBW7* gene leading to anti-apoptotic MCL1 stabilization and resistance to Bcl-2 inhibitor in T-cell acute lymphoblastic leukemia. *FBW7* gene mutation causes the accumulation of oncogenic driver molecules such as c-Myc, c-Jun, Notch-1, which then results in the activation of cellular proliferation signal pathways. On the other hand, the loss-of-functional mutation of *FBW7* gene enhances the stabilization of MCL1, one of the major anti-apoptotic molecules which is frequently overexpressed in T-cell acute lymphoblastic leukemia (T-ALL). In the clinical settings, increased expression of MCL1 in FBW7-deficient T-ALL cells induces the therapeutic resistance to the BH3 mimetic ABT-737, a pan-inhibitor of the Bcl-2 family of anti-apoptotic proteins [211–213]. Note that red arrows indicate up-regulation, whereas blue bars show the inhibitory effect. In addition, the yellow particles in the mitochondrion correspond to cytochrome *c*

It is notable that FBW7 holds the promising therapeutic target to eliminate CSCs population. Because FBW7 is an essential component of an ubiquitin ligase responsible for the degradation of oncogenic c-Myc [179, 192], low level of FBW7 expression in the tumor microenvironment is associated with poor prognosis in breast cancer patients [206]. In contrast, the elevated serum levels of CCL2 have been shown to be associated with poor prognosis in breast cancer patients [206]. FBW7 depletion in BM-derived stromal cells (BMSCs) results in the accumulation of Notch-1 intracellular domain (NICD1) and increased secretion of CCL2, which in turn promotes recruitment of monocytic myeloid-derived suppressor cells (Mo-MDSCs) and tumor-associated macrophages (TAMs). Thus, the regulation of FBW7 is expected to exhibit anti-metastatic function through the regulation of the interaction between Notch-1 and CCL2 in tumor stroma composed of F4/F80-positive TAMs and Ly6C-positive MDSCs [206–208]. Furthermore, the loss-of-functional mutation of *FBW7* gene results in the increased stabilization of MCL1, one of the major anti-apoptotic molecules which is frequently overexpressed in T-ALL [208] (Fig. 7). That is why down-regulation of MCL1 in FBW7-deficient T-ALL cells is expected to overcome the therapeutic resistance to the BH3 mimetic ABT-737, which is a pan-inhibitor of the Bcl-2 family of anti-apoptotic proteins including BCL2, BCL_XL_, and BCL_W_. Notably, it has been shown that sorafenib (BAY 43-9006), one of the widely-used tyrosine kinase inhibitors for the treatment of renal cell carcinoma [209, 210], exhibits the synergistic therapeutic effect with ABT-737 against FBW7-deficient T-ALL cells [211–213].

**Fig. 8:**
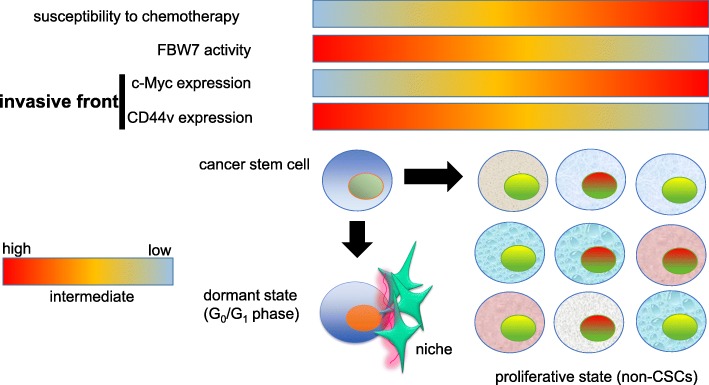
Plasticity of cancer stem-like cells between dormant and proliferative conditions in terms of c-Myc regulation by FBW7. CSCs exhibit the plasticity depending on the tumor microenvironment, which is why CSCs can efficiently escape from the attack of the anti-tumor combined modality therapy compared with non-CSCs. Given that c-Myc undergoes ubiquitin-proteasome-dependent degradation by FBW7, CSCs highly expressing CD44v and FBW7 and lowly expressing c-Myc tend to be quiescent (G_0_/G_1_ dormant phase). ROS-induced activation of canonical Wnt/β-catenin signal pathway is responsible for up-regulation of c-Myc at the invasive front enriched in CSCs [118–120]. Note that, even under the proliferative conditions, non-CSCs show the genetic and/or epigenetic heterogeneity

From the perspectives of therapeutic strategies targeting CSCs, FBW7 expression has been reported to be highly expressed in CSC [214, 215]. As compared with non-CSCs, CSCs tend to exhibit quiescence (G_0_/G_1_ phase) [216–218]. The quiescent property of CSCs has long been believed to reduce their susceptibility to chemotherapy, which is consistent with the low level of c-Myc expression (Fig. 8). For instance, mitotic inhibitors characterized by paclitaxel and vincristine preferentially eliminate proliferating non-CSCs during M phase of the cell cycle. Anti-metabolite agents characterized by 5-fluorouracil (5-FU), 6-mercaptopurine, and methotrexate induce genotoxic stress during S phase [219, 220]. Topoisomerase inhibitors such as irinotecan (CPT-11) and etoposide (VP-16) interrupts the separation of DNA strands during DNA replication and transcription [221, 222]. However, these drugs show anti-tumor effects only when cancer cells are under proliferative conditions. By striking contrast, CSCs in the dormant state (G_0_/G_1_ quiescent phase of the cell cycle) are refractory to such conventional anti-tumor drugs, the action points of which are largely dependent on the operation of the cell cycle. That is why the loss-of-functional mutations of *FBW7* gene trigger“awakening” of dormant CSCs in the niche with up-regulation of c-Myc. Notably, the inversed expression pattern between CD44v and c-Myc is significant at the invasive front enriched in CSCs of several kinds of solid tumors including gastric, breast and nasopharyngeal carcinomas due to ROS-mediated canonical Wnt/β-catenin signal activation [118, 120, 223, 224] (Fig. 8).

Taken together, this “locked-out” therapeutic strategy with FBW7 inhibition with the conventional anti-tumor chemicals to drive CSCs out of G_0_/G_1_-phased quiescent conditions is potentially effective to overcome the low susceptibility of CSCs to anti-tumor drugs, but its possible adverse events will need to be carefully investigated [119, 185, 218]. There is a possibility that the inhibition of FBW7 and consequent up-regulation of c-Myc might promote tumor cell proliferation and aggressive metastasis before the combined modality therapy is able to eliminate CSCs. By striking contrast, the“locked-in” therapeutic strategy is expected to prevent further cancer development as well as latent relapse due to the presence of the persistent MRD, only when the proliferative capacity of CSCs remains suppressed for the long lifetime of the patient.

The screening of the suitable small molecular-sized chemicals and/or the biologically-effective inhibitors targeting Myc is expected to be very difficult given the localization of c-Myc in the nucleus and the absence of its deep surface-binding pocket [225]. To put it simply, the direct inhibition of c-Myc remains a central challenge in the discipline of ligand discovery. On the other hand, there have been several reports suggesting the potential therapeutic strategy targeting Myc mRNA/ protein both in vitro and in vivo. Firstly, Omomyc is known to be a Myc-derived bHLH-Zip domain obtained by the substitution of the four amino acids in the Myc zipper which have been identified as to inhibit the binding of c-Myc to E-box region [226]. Omomyc has been reported to form the heterodimer with wild-type c-Myc, thereby interfering with the formation of Myc/Max heterodimers and suppressing the binding of c-Myc to E-box elements. As a result, Omomyc suppresses the activation of E-box promoter elements by Myc/Max and inhibits cancer cell proliferation [226, 227]. The pro-apoptotic potential of Omomyc is recognized exclusively in cancer cells expressing enhanced level of c-Myc, which suggests that the ability of Omomyc to promote the apoptosis seems to largely depend on the level of c-Myc. Secondly, it is widely accepted that the inhibition of the bromodomain and extraterminal (BET) protein BRD4 by JQ1 results in the suppression of *c-Myc* transcription [228]. JQ1 is a thieno-triazolo-1,4-diazepine which displaces BET bromodomains from chromatin by competitively binding to the acetyl-lysine recognition pocket. Mediator is known to be a co-activator complex which interacts with transcriptional factors and participates in the recruitment and the activation of RNA polymerase II (Pol II) [229]. The location of super-enhancers with the exceptional level of BRD4 and Mediator provides the molecular basis for the hypersensitivity of specific genes to JQ1-mediated transcriptional suppression [230, 231]. However, because c-Myc is mainly localized in the nucleus and does not have a deep surface-binding pocket, the identification of the small molecular-sized and biologically-effective chemicals directly targeting Myc seems to be challenging [225]. That is mainly why DR therapeutic strategy holds much promise as shown in Table 1 [75, 76, 85, 117, 130, 232–236].

## Conclusions

Since the discovery of c-Myc in Burkitt lymphoma about 40 years ago, the numerous research papers have been reported to elucidate the pathophysiological roles of Myc in cancer cells. Indeed, Myc has several functions in tumor cells related to cellular proliferation and metabolic reprogramming. Furthermore, as the concept of CSCs attracts much attention as the highly tumorigenic subpopulation of tumor cells existing at the top of the hierarchical tumor cellular society, the specific functions of Myc in CSCs and the novel therapeutic strategies have been recently uncovered. It is highly likely that the persistent tumor cells after chemotherapy consisting of MRD highly express Myc as compared with cancer cells which are susceptible to the conventional treatment. Therefore, the challenging research is warranted to discover the novel therapeutic target machinery regulated by Myc family and to realize the precision medicine in the near future given the intra-tumoral heterogeneity of Myc expression pattern.

## Abbreviations

ACR: Acyclic retinoid
AMPK: AMP-activated protein kinase
ASCL1: Achaete-scute homolog 1
ATRA: All-*trans* retinoic acid
BET: Bromodomain and extraterminal
bHLH: Basic helix-loop-helix
BM: Bone marrow
BMSCs: BM-derived stromal cells
BPTES: BIS-2-[5–phenylacetamido-1, 2, 4-thiadiazol-2-yl] ethyl sulfide
CD44s: CD44 standard isoform
CD44v: CD44 variant isoform
Cdc4: Cell division control protein 4
ChIP: Chromatin immunoprecipitation
CSCs: Cancer stem-like cells
DKO: Double-knockout
DLBCL: Diffuse large B-cell lymphoma
DR: Drug-repositioning
EMT: Epithelial-mesenchymal transition
ERK: Extracellular signal-regulated kinase
ES: Embryonic stem
ESRP1: Epithelial splicing regulatory protein 1
EZH2: Enhancer of zeste homolog 2
FAO: Fatty acid β-oxidation
FBW7: F-box and WD repeat domain-containing 7
GEMMs: Genetically engineered mouse models
GLS: Glutaminase
GLUT: Glucose transporter
GSH: Reduced form of glutathione
GSSG: Oxidative form of glutathione
H3K27ac: Histone H3 lysine 27 acetylation
H3K4me1: Histone H3 lysine 9 monomethylation
HCC: hepatocellular carcinoma
HDAC: Histone deacetylase
HIF: Hypoxia-inducible transcription factor
HK: Hexokinase
HSCs: Hematopoietic stem cells
ICD: Intracellular domain
IFN-γ: Interferon-γ
IL-6: Interleukin-6
iPS: Induced pluripotent stem
JAK: Janus kinase
LDH: Lactate dehydrogenase
LIF: Leukemia inhibitory factor
MAPK: Mitogen-activated protein kinase
Max: Myc-associated protein X
MDSCs: Myeloid-derived suppressor cells
MEFs: Mouse embryonic fibroblasts
miRNA: MicroRNA
MRD: Minimal residual disease
mTOR: Mammalian target of rapamycin
NADPH: Reduced form of nicotinamide adenine dinucleotide phosphate
NICD1: Notch-1 intracellular domain
NSCs: Neural stem cells
PK: Pyruvate kinase
PKM1/2: PK isoenzyme type M1/2
PMN: Proximal Myc network
Pol II: RNA polymerase II
PRC2: Polycomb repressive complex 2
PTEN: Phosphatase and tensin homologue deleted on chromosome 10
RBP-J: Recombination signal binding protein for immunoglobulin κ J region
ROS: Reactive oxygen species
SCF: SKP1-CUL1-F-box protein
SCLC: Small-cell lung cancer
Skp1: S-phase kinase-associated protein 1
STAT: Signal transduction and activator of transcription
T-ALL: T-cell acute lymphoblastic leukemia
TAMs: Tumor-associated macrophages
TCGA: The Cancer Genome Atlas
TENs: Tumor-entrained neutrophils
TNBC: Triple-negative breast cancer
UPS: Ubiquitin proteasome system
αENaC: α subunit of the epithelial sodium channel
α-KG: α-ketoglutarate
