# Correction to: Emerging roles of Myc in stem cell biology and novel tumor therapies

**DOI:** 10.1186/s13046-018-0964-3

**Published:** 2018-11-27

**Authors:** Go J. Yoshida

**Affiliations:** Department of Pathological Cell Biology, Medical Research Institute, Tokyo Medical and Dental University, 1-5-45 Yushima, Bunkyo-ku, Tokyo, 113-8510 Japan

## Correction

In the publication of this article [1] there are three errors. The author would like to apologize to all the readers and researchers who contributed to the related works. This has now been included in this correction.

1. The error: The author categorically stated in abstract that “N-Myc-driven neuroendocrine tumors tend to highly express NEUROD1,” but there is a controversy as to whether or not neuroendocrine lung cancer cells highly express NEUROD1. Not a few researchers believe that there is no clear correlation between N-Myc and NEUROD1. ASCL1 and NEUROD1 are required for the survival and proliferation of SCLCs. Importantly, ASCL1 but not NEUROD1 directly regulates well-known SCLC-related oncogenes and is required for tumor formation in vivo of GEMMs.

Should instead read: N-Myc and p53 exhibit the co-localization in the nucleus and alter p53-dependent transcriptional responses which are necessary for DNA repair, anti-apoptosis, and lipid metabolic reprogramming. NCYM protein stabilizes N-Myc, resulting in the stimulation of Oct4 expression, while Oct4 induces both N-Myc and NCYM via direct transcriptional activation of N-Myc.

2. The error: After the publication of this review, the author noticed the rate of N-Myc amplification was incorrectly written.

Should instead read: Myc has been reported to be amplified in 15–20% of small-cell lung cancer (SCLC) tissues, while N-Myc is amplified in less than 5% of those patients [60, 61]. Myc amplification is associated with poor clinical prognosis and therapeutic response to chemotherapy [62, 63].

3. The error: In addition, the author had incorrectly used the term ‘N-Myc’ in the paragraph which describes the pathological characteristics and therapeutic response of genetically engineered mouse model of small cell lung cancer.

Should instead read: In genetically engineered mouse models (GEMMs), it has been shown that murine c-Myc-driven SCLC expresses high level of NEUROD1, which is a key transcriptional factor for the survival and proliferation of neuroendocrine tumor cells [55, 67]. Based on in situ immunostaining patterns for achaete-scute homolog 1 (ASCL1) and NEUROD1, it is proposed that c-Myc-driven cancer cells emerge among ASCL1-positive precursor cells, and these early-staged cancer cells initially exhibit classic morphology. With the passage of time, it seems likely that tumor cells change into an ASCL1 (low)/NEUROD1 (high) expression pattern which is coincident with the appearance of variant morphology phenotype in GEMMs [67]. Because the overexpression of NEUROD1 has been linked to the development of metastases and aggressive SCLC phenotypes [68], it has been suggested that c-Myc activation results in the variant characteristics via NEUROD1 signal activation. From the therapeutic perspectives, c-Myc expression levels, the neuroendocrine-low expression profile, and variant pathohistopathology are all expected to serve as the useful biomarkers to predict the sensitivity to Aurora kinase inhibition in the clinical settings. It has been shown that Aurora kinase inhibition is highly likely to improve the chemotherapy response in vivo, which strongly suggests that the patients with c-Myc-amplified SCLCs exhibit the significant clinical benefit from the first-line therapy with Aurora kinase inhibitors in combination with the conventional chemotherapy [67, 69, 70].
